# Generation of human androgenetic induced pluripotent stem cells

**DOI:** 10.1038/s41598-020-60363-1

**Published:** 2020-02-27

**Authors:** Na Young Choi, Jin Seok Bang, Yo Seph Park, Minseong Lee, Han Sung Hwang, Kisung Ko, Soon Chul Myung, Natalia Tapia, Hans R. Schöler, Gwang Jun Kim, Kinarm Ko

**Affiliations:** 1 Department of Stem Cell Biology, School of Medicine, Konkuk University, Seoul, 05029 Republic of Korea; 2 Center for Stem Cell Research, Institute of Advanced Biomedical Science, Konkuk University, Seoul, 05029 Republic of Korea; 3 Department of Stem Cell Research, TJC Life Research and Development Center, TJC Life, Seoul, 06698 Republic of Korea; 4 Department of Obstetrics and Gynecology, Research Institute of Medical Science, Konkuk University School of Medicine, Seoul, 05030 Republic of Korea; 5 Department of Medicine, College of Medicine, Chung-Ang University, Seoul, 06974 Republic of Korea; 6 Department of Urology, Chung-Ang University College of Medicine, Seoul, 06974 Republic of Korea; 7 Institute of Biomedicine of Valencia, Spanish National Research Council, Jaime Roig 11, 46010 Valencia, Spain; 8 Department of Cell and Developmental Biology, Max Planck Institute for Molecular Biomedicine, 48149 Münster, Germany; 9 Medical Faculty, University of Münster, 48149 Münster, Germany; 10 Department of Obstetrics and Gynecology, Chung-Ang University Hospital, Chung-Ang University College of Medicine, Seoul, 06974 Republic of Korea; 11 Research Institute of Medical Science, Konkuk University, Seoul, 05029 Republic of Korea

**Keywords:** Imprinting, Induced pluripotent stem cells

## Abstract

In humans, parthenogenesis and androgenesis occur naturally in mature cystic ovarian teratomas and androgenetic complete hydatidiform moles (CHM), respectively. Our previous study has reported human parthenogenetic induced pluripotent stem cells from ovarian teratoma–derived fibroblasts and screening of imprinted genes using genome-wide DNA methylation analysis. However, due to the lack of the counterparts of uniparental cells, identification of new imprinted differentially methylated regions has been limited. CHM are inherited from only the paternal genome. In this study, we generated human androgenetic induced pluripotent stem cells (AgHiPSCs) from primary androgenetic fibroblasts derived from CHM. To investigate the pluripotency state of AgHiPSCs, we analyzed their cellular and molecular characteristics. We tested the DNA methylation status of imprinted genes using bisulfite sequencing and demonstrated the androgenetic identity of AgHiPSCs. AgHiPSCs might be an attractive alternative source of human androgenetic embryonic stem cells. Furthermore, AgHiPSCs can be used in regenerative medicine, for analysis of genomic imprinting, to study imprinting-related development, and for disease modeling in humans.

## Introduction

The contribution of both the maternal and paternal genomes is required for normal development. The maternal and paternal genetic contributions are important in early embryonic development and placental development, respectively^1,2^. A uniparental embryo with two maternal genomes is termed parthenogenetic, whereas that with two paternal genomes is termed androgenetic. Uniparental embryos fail to develop^3,4^.

Recent studies have reported the derivation of human parthenogenetic embryonic stem cells (PgESCs) from parthenogenetic embryos^5–8^. These studies have determined the methylation status and expression levels of imprinted genes in human PgESCs. Human androgenetic embryonic stem cells (AgESCs) can be generated from androgenetic embryos^9^. Human AgESCs have androgenetic imprinting status because of the lack of the maternal genome. Bisulfite sequencing analysis of the methylation status of human AgESCs revealed that paternally imprinted genes (*SNRPN* and *KCNQ1*) were hypomethylated, whereas maternally imprinted genes (*H19* and *MEG3*) were hypermethylated^9^. These findings indicate that the androgenetic identity of uniparental embryonic stem cells (ESCs) can be confirmed by determining the methylation status of imprinted genes. Although human AgESCs are considered attractive cell lines, their generation poses ethical issues because of the use of human eggs and the destruction of human embryos.

Although they are rare, parthenogenesis and androgenesis occur naturally in humans. Mature cystic ovarian teratomas, also known as dermoid cysts, arise from parthenogenetic activation of oocytes^10,11^. Hydatidiform moles are abnormal pregnancies and can be classified as complete hydatidiform moles (CHM) or partial hydatidiform moles (PHM) depending on their genetic origin^12,13^. Most CHM have an entirely paternal origin and lack the maternal genome. They may arise from the fertilization of oocytes without nucleus by a single sperm or two sperms^14^. About 90% of CHM have the 46, XX karyotype, whereas ~10% have the 46, XY karyotype^12,15^. PHM have a triploid genome (69, XXY; 69, XXX; or 69, XXY) and result from fertilization of an egg by two sperms^12^.

Human induced pluripotent stem cells (HiPSCs) have been derived from various fibroblasts^11,16–20^. Previously, we generated human parthenogenetic induced pluripotent stem cells (PgHiPSCs) from mature cystic ovarian teratoma–derived human parthenogenetic fibroblasts (PgFibs) for screening of imprinted genes^21^. However, due to the lack of the counterparts of uniparental cell lines, comprehensive study of genomic imprinting has been limited. In this study, we report for the first time generation of human androgenetic induced pluripotent stem cells (AgHiPSCs) from CHM-derived fibroblasts. AgHiPSCs can be used for comprehensive methylation analysis along with PgHiPSCs, as well as in regenerative medicine, research on imprinting-related development and disease modeling.

## Results

### Derivation of human androgenetic fibroblasts

The CHM types have been classically described as “bunch of grapes”, “snowstorm”, or “granular”^22–24^. We isolated fibroblasts from a CHM resembling a bunch of grapes (Supplementary Data Fig. S1a) using collagenase/hyaluronidase to dissociate the tissue into single cells, as described for mouse and human mammary gland tissues^25,26^. The androgenetic fibroblasts (AgFibs) exhibited morphology typical for human fibroblasts (Supplementary Data Fig. S1b).

To confirm the androgenetic imprinting status of AgFibs, we examined the methylation status in the differentially methylated regions (DMRs) of the maternally imprinted gene, *MEST*. We previously used bisulfite sequencing of the DMRs of *MEST* to identify the imprinting status of PgFibs and PgHiPSCs^21^. As shown in Supplementary Data Fig. S1c, *MEST* was hypermethylated in PgFibs, which were used as a control, but not in AgFibs. As expected, biparental fibroblasts showed somatic imprinting patterns in *MEST* (Supplementary Data Fig. S1c). These results confirm the androgenetic identity of AgFibs.

### Generation and characterization of human androgenetic induced pluripotent stem cells

**Figure 1:**
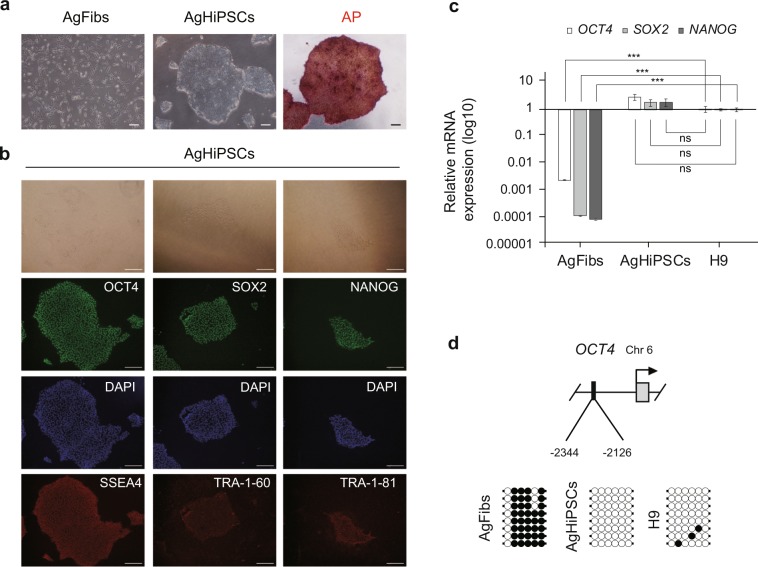
Characterization of human androgenetic induced pluripotent stem cells. (**a**) Morphology of AgFibs and AgHiPSCs, and alkaline phosphatase (AP) staining of AgHiPSCs. Scale bars = 100 μm. (**b**) Immunocytochemistry for pluripotency markers. Scale bars = 100 μm. (**c**) qRT-PCR analysis of the expression of *OCT4*, *SOX2*, and *NANOG* in AgFibs and AgHiPSCs, compared with the H9 positive control. The expression levels in H9 were set to 1. Data are shown as mean ± SEM (n = 3). Significance of the differences between cell types was determined by *t*-test, and *p*-values < 0.001 are indicated with ***. Ns means not significant. (**d**) Bisulfite sequencing analysis of *OCT4* promoter regions in AgFibs, AgHiPSCs, and H9 cells. Each line represents a separate clone. Black and white circles represent hypermethylated and hypomethylated CpGs, respectively.

**Figure 2:**
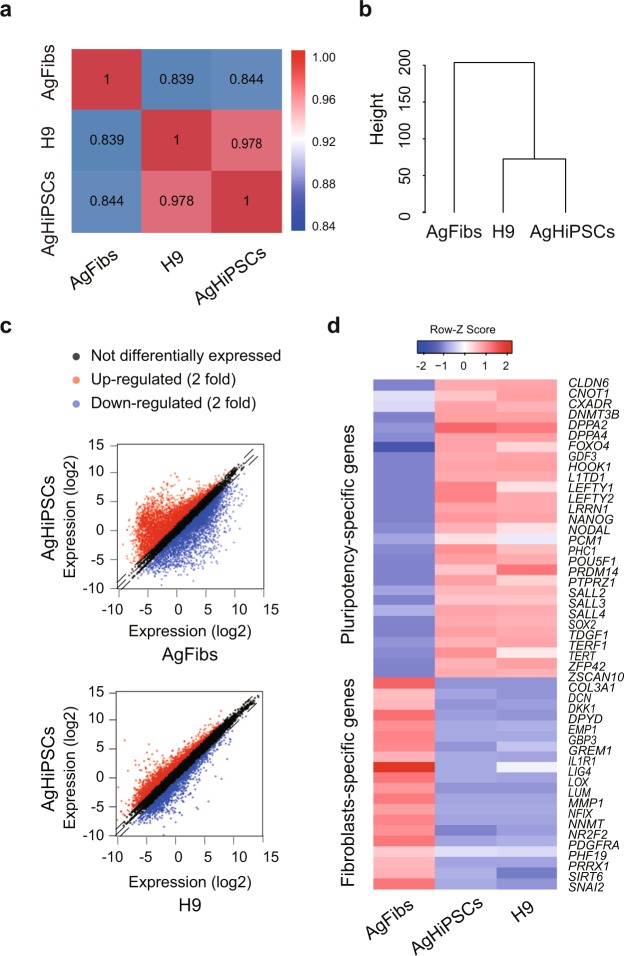
Global expression analysis in human androgenetic induced pluripotent stem cells. (**a**) Heatmap showing Pearson correlation coefficients among samples. (**b**) Hierarchical clustering of human pluripotent stem cells and their parental fibroblasts based on transcriptome analysis. (**c**) Scatter plots comparing global gene expression patterns determined by RNA sequencing. (**d**) Expression levels of human pluripotency-specific genes and fibroblasts-specific genes in AgFibs, AgHiPSCs, and H9 cells.

To generate an AgHiPSC line, AgFibs were infected with lentiviruses expressing reprogramming factors *OCT4* (also known as *POU5F1*), *SOX2*, *KLF4*, and *cMYC*^17^. AgHiPSCs showed typical ESC-like morphology and stained positive for alkaline phosphatase (Fig. 1a). We confirmed the expression of the pluripotency-specific markers OCT4, SOX2, NANOG, SSEA4, TRA-1–60, and TRA-1–81 in AgHiPSCs by immunocytochemical analysis (Fig. 1b). AgFibs and human ESCs (H9) were used as negative and positive controls, respectively (Supplementary Data Fig. S2). The expression levels of *OCT4*, *SOX2*, and *NANOG* in AgHiPSCs were similar to those in H9 as revealed by quantitative real-time PCR (qRT-PCR) and RT-PCR analysis (Fig. 1c and Supplementary Data Fig. S3). Bisulfite sequencing analysis showed that *OCT4* was hypomethylated in AgHiPSCs and H9 cells, but was hypermethylated in AgFibs (Fig. 1d). We performed RNA-sequencing analysis to compare global gene expression. H9 cells were used as control. To investigate the dependence between two sets of data, we calculated using Pearson correlation. We found positive Pearson correlation coefficients values between samples. The correlation analysis between these samples is represented in Fig. 2a. Hierarchical clustering showed that the global gene expression profile of AgHiPSCs was similar to that of H9 cells (Fig. 2b). As expected, scatter plots showed high correlation between AgHiPSCs and H9 cells but no correlation between AgHiPSCs and AgFibs (Fig. 2c). The expression of pluripotency-specific genes and fibroblast-specific genes is shown as a heat map in Fig. 2d. All these cell lines had a normal karyotype (46, XX; Supplementary Data Fig. S5). Short tandem repeat analysis confirmed the same genetic identity between AgFibs and AgHiPSCs (Supplementary Data Fig. S6).

**Figure 3:**
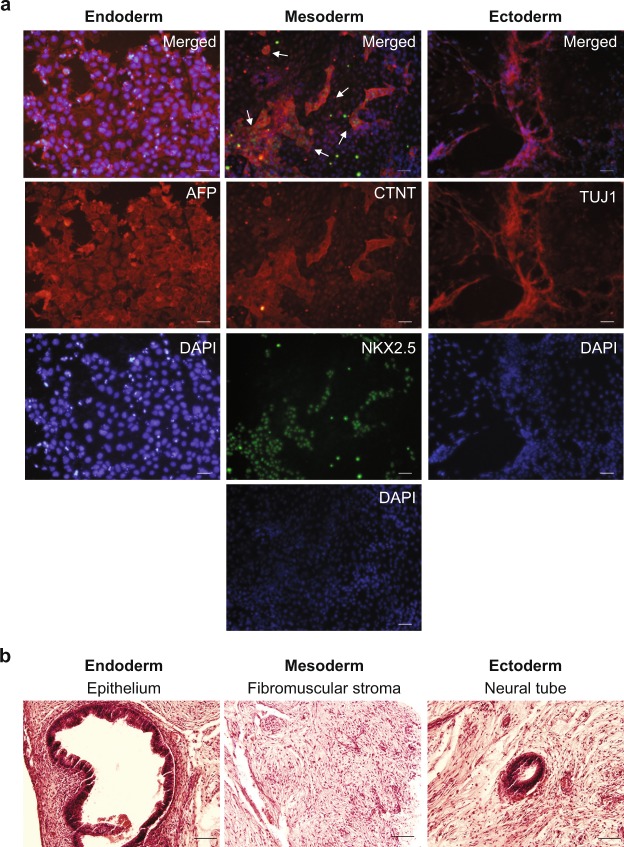
*In vitro* and *in vivo* differentiation of human androgenetic induced pluripotent stem cells. (**a**) Immunocytochemistry of AgHiPSC-derived cells in the three germ layers after differentiation into embryoid bodies: endoderm (AFP), mesoderm (CTNT and NKX2.5), and ectoderm (TUJ1). Scale bars = 200 μm. (**b**) Teratoma stained with hematoxylin and eosin. Scale bars = 100 μm.

AgHiPSCs could be differentiated into three germ layers *in vitro* through embryoid body (EB) formation and *in vivo* through teratoma formation. We confirmed the expression of layer-specific gene markers in the endoderm (*AFP*, *GATA4*, and *SOX17*), mesoderm (*MSX* and *BRACHYURY*), and ectoderm (*PAX6*) of differentiated EBs by using RT-PCR analysis (Supplementary Data Fig. S7). In addition, these cells were positive for the markers of endoderm (AFP), mesoderm (NKX2.5 and CTNT), and ectoderm (TUJ1) in immunocytochemical analysis (Fig. 3a). We transplanted AgHiPSCs subcutaneously into NOD/SCID mouse. Three months after injection, we confirmed the formation of teratoma containing the three germ layers (Fig. 3b). These results indicate that AgHiPSCs are pluripotent and capable of differentiation.

### Genome-wide single-nucleotide polymorphism analysis of human androgenetic induced pluripotent stem cells

**Figure 4:**
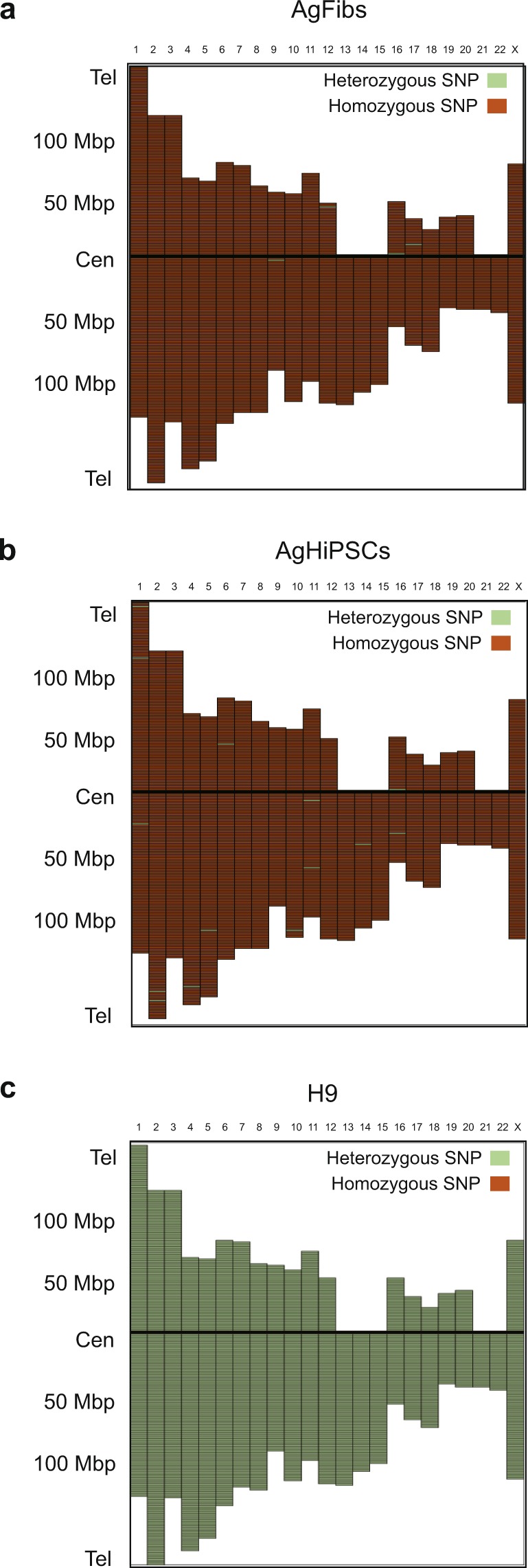
Genomic homozygosity patterns in AgFibs, AgHiPSCs, and H9 cells determined by genome-wide SNP analysis. SNP marker distance from the centromere (Cen) to the telomere (Tel) along each chromosome of (**a**) AgFibs, (**b**) AgHiPSCs, and (**c**) H9 cells.

To measure the recombination rate in AgHiPSCs, we used single-nucleotide polymorphism (SNP) analysis. AgFibs and AgHiPSCs were homozygous at random SNP markers along each chromosome, whereas H9 cells were heterozygous (Supplementary Data Fig. S9 and Fig. 4). These results show that the genotype of the reprogrammed cells was identical to that of their parental fibroblasts.

### Characterization of the known imprinted genes in human androgenetic induced pluripotent stem cells

**Figure 5:**
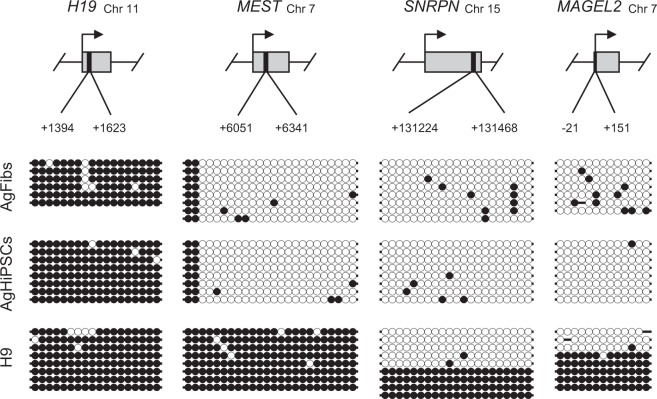
Characterization of the known imprinted genes in AgHiPSCs. DNA methylation status of known paternally and maternally imprinted genes (*H19*, *MEST*, *SNRPN*, and *MAGEL2*) in AgFibs, AgHiPSCs, and H9 was analyzed by bisulfite sequencing. Each line represents a separate clone. Black and white circles represent hypermethylated and hypomethylated CpGs, respectively.

To confirm the androgenetic imprinting status of androgenetic cells, we compared the DNA methylation status in the DMRs of the known imprinted genes by using bisulfite sequencing. The paternally imprinted gene *H19* was hypermethylated in AgFibs and AgHiPSCs, whereas the maternally imprinted genes *MEST*, *SNRPN*, and *MAGEL2* were hypomethylated (Fig. 5). *SNRPN* and *MAGEL2* had somatic imprinting status in H9 cells, whereas *H19* and *MEST* were hypermethylated. Epigenetic instability of the imprinted genes *H19* and *MEST* in human ESCs was previously reported^21,27–29^. These results demonstrate the androgenetic imprinting status of AgHiPSCs.

## Discussion

In rare cases, parthenogenesis and androgenesis occur spontaneously in humans. The use of the respective uniparental tissues does not require destruction of viable human embryos, which makes uniparental embryos an attractive alternative source of pluripotent stem cells.

CHM have only paternal chromosomes. In this study, we established AgFibs from CHM and used them to generate a homogenous population of AgHiPSCs by transduction of transcription factors. AgFibs were reprogrammed to AgHiPSCs despite having only the paternal genome. AgHiPSCs showed characteristic features of typical human ESCs in pluripotency, gene expression profile, *in vitro* differentiation potential, and *in vivo* teratoma formation. Several studies have determined the degree of homozygosity of human PgESCs^30–33^ and human AgESCs^9^ by SNP analysis, which can be used to determine the ratio of heterozygous alleles. Our SNP analysis showed 99.53% homozygosity in AgFibs, 99.48% in AgHiPSCs, and 72.72% in H9 cells. According Ding *et al*.^9^, human AgESCs were 99.39% homozygous, in contrast to 72.85% homozygosity in H9 cells. These data confirmed that our androgenetic cell lines were homogeneous.

Our previous study reported that the parthenogenetic imprinting status of imprinted genes (*H19* and *MEST*) was maintained in parthenogenetic cell lines during the acquisition of pluripotency^21^. In the present study, we confirmed that the androgenetic imprinting status in DMRs of known imprinted genes (*H19*, *MEST*, *SNRPN*, and *MAGEL2*) was maintained after reprogramming. Human AgESCs have androgenetic imprinting patterns in the DMRs of the *H19*, *SNRPN*, and *MEG3* genes^9^. In particular, the DMRs of *SNRPN* used to verify the methylation status of AgHiPSCs in our study overlap with those used by Ding *et al*.^9^ to confirm the androgenetic imprinting status in human AgESCs. Therefore, our data are in agreement with those of previous studies.

Genomic imprinting has attracted much attention because it is linked to human genetic diseases. Most genetic disorders can be caused by dysfunction of imprinted genes and chromosomal regions. Typically, Beckwith–Wiedemann syndrome (hypermethylation at *H19*) and Russell–Silver syndrome (hypomethylation at *H19*) are caused by dysregulation of imprinting control in a region of chromosome 11p15^34,35^. Prader–Willi syndrome (PWS) and Angelman syndrome (AS) are neurogenetic disorders caused by genetic defects in one or more imprinted genes on chromosome 15q11–q13^36^. Our previous study reported that PgHiPSCs and HiPSCs were differentiated into neural stem cells and the parthenogenetic imprinting status of the PWS/AS-related region was maintained during reprogramming and neural differentiation^37^. Because genetic disorders have diverse mechanisms of mutagenesis, it is important to obtain samples containing cells with uniparental chromosomes in order to identify the causative genes. Since AgHiPSCs can be differentiated into all somatic cell lineages, they can be used for a variety of applications and could be an important tool to study genomic imprinting of imprinted genes related to development and disorders.

## Methods

### Ethics statement

This study is approved by the Institutional Review Board of Konkuk University Medical Center (KUH 1040045). During the informed consent process, prospective research participants were given comprehensive information about purpose of the research, procedures, and confidentiality. There are no anticipated risks or benefits for participation. Participation is voluntary and may decide to discuss participation with family or friends. Human CHM tissue was collected from consenting donors at Konkuk University Medical Center. All human experiments were performed in accordance with relevant guidelines and regulations. Animal experiments were approved by Institutional Animal Care and Use Committee of Konkuk University. Animal handling was in accordance with the respective institutional animal protection guidelines.

### Derivation of human androgenetic fibroblast cells

CHM were washed with Dulbecco’s Phosphate Buffered Saline (DPBS, Welgene). Collagenase/hyaluronidase (10X stock solution) was purchased from StemCell Technologies. CHM were dissociated into a single-cell suspension by incubating them in 1X collagenase/hyaluronidase and DNase (1 mg/mL) at 37 °C until all large tissue fragments were digested (typically in 50 min). Cell suspension was filtered through a 40 µm cell strainer to remove clumps. Cells were grown in BIOAMF-2 complete medium (Biological Industries). The cell culture medium was changed every other day.

### Generation of human androgenetic induced pluripotent stem cells

To prepare the lentivirus, 293T cells purchased from the American Type Culture Collection (ATCC Cat# CRL-3216) were transfected with 15 μg lentiviral vector^38^ by using the Infect transfection reagent (iNtRON) according to the manufacturer’s instructions. Supernatants were collected 48 h after transfection and filtered through a 0.45 μm filter unit. Human androgenetic fibroblasts were plated at 20,000 cells per well in a 6-well plate coated with Matrigel and infected with the lentivirus supplemented with 4 μg/ml polybrene (Sigma-Aldrich). Two days after transduction, medium was changed to TeSR-E7 medium (StemCell Technologies). The colonies were picked up and transferred into a 6-well plate coated with Matrigel (Corning) containing mTeSR1 medium (StemCell Technologies).

### Alkaline phosphatase staining

Alkaline phosphatase (AP) staining was performed using an AP staining kit (Stemgent). Cells were fixed with fixation solution for 5 min and stained with AP substrate solution for 15 min at room temperature.

### Immunocytochemistry

Cells were fixed with DPBS containing 4% paraformaldehyde (Sigma-Aldrich) for 10 min at room temperature, washed three times with DPBS, permeabilized for 15 min with DPBS containing 0.5% Triton X-100 (Sigma-Aldrich), and blocked for 1 h with 1% BSA (Gibco) in DPBS, followed by incubation with antibodies from a StemLight Pluripotency Antibody Kit (1:200, Cell Signaling Technology) or antibodies against alpha-fetoprotein (1:200, AFP, R&D Systems), NKX2.5 (1:200, Cell Signaling Technology), cardiac troponin T (1:200, CTNT, Abcam), and neuron-specific class III beta-tubulin (1:200, TUJ1, Millipore) overnight at 4 °C. After washing in DPBS, cells were incubated with secondary antibodies (1:1000, against rabbit IgG and mouse IgG from Cell Signaling Technology or mouse IgM from Invitrogen) for 1 h at room temperature and were stained with 0.5 μg/ml DAPI (Invitrogen) to detect the nuclei.

### PCR and quantitative real-time PCR

Total RNA was isolated using a miRNeasy Mini Kit (Qiagen). Genomic DNA was removed from total RNA using an RNase-Free DNase set (Qiagen). cDNA was synthesized using a High Capacity cDNA Reverse Transcription Kit (Applied Biosystems). Quantitative real-time PCR (qRT-PCR) was performed with a Real-Time PCR system (Applied Biosystems) and all reactions were run in triplicate. Primer sequences were described in a previous study^21^.

### RNA sequencing

Whole-transcriptome library construction and data analysis were carried out. Paired-end RNA sequencing was conducted once in each sample using an Illumina Hiseq 2500 system (Illumina Inc.). Illumina Casava version 1.8.2 software was used for basecalling. The rRNA ratio was checked using Fastq screen version 0.11.1. RNA sequencing reads were aligned to the reference genome (human hg19) using TopHat version 2.0.13 (http://tophat.cbcb.umd.edu)^39^ and generated bam files. Abundance was calculated as fragments per kilobase of transcript per million (FPKM) values. Differentially expressed genes were selected (fold change ≥2) and analyzed using Cufflinks version 2.2.0 (http://cufflinks.cbcb.umd.edu)^39^. The RNA sequencing data have been deposited in the Gene Expression Omnibus (GEO) under the accession number GSE141906 (https://www.ncbi.nlm.nih.gov/geo/query/acc.cgi?acc=GSE141906).

### *In vitro* differentiation

For EB formation, AgHiPSCs were harvested using ReLeSR (StemCell Technologies), and cell clumps were resuspended in DMEM/F12 (Corning) with 20% Knockout serum replacement (Gibco), 2 mM L-glutamine (Welgene), 1% non-essential amino acids (Gibco), 1% penicillin-streptomycin (Welgene), and 50 μM β-mercaptoethanol (Gibco). After 14 days, RNA was isolated from EBs. For monolayer differentiation, AgHiPSCs were cultured in the three germ layer differentiation medium as previously described^40,41^. The cell culture medium was changed every other day.

### *In vivo* teratoma formation

Undifferentiated AgHiPSCs were harvested and collected into a tube. The cell pellets were resuspended in a 1:1 mixture of mTeSR and Matrigel. AgHiPSCs were injected subcutaneously into NOD/SCID mouse. After 3 months, teratomas were surgically dissected from the mice, fixed in Bouin’s solution (Sigma), embedded in paraffin and stained with hematoxylin and eosin.

### Genomic DNA and bisulfite treatment

Genomic DNA was isolated using a G-spin Total DNA Extraction Kit (iNtRON). One μg of genomic DNA was bisulfite converted an EpiTect Bisulfite Kit (Qiagen). Thirty-five μL of DNA protect buffer and 85 μL of Bisulfite Mix were added to maximum 20 μL genomic DNA in PCR tube. The mixtures were heated for 5 min at 95 °C, 25 min at 60 °C, 5 min at 95 °C, 85 min at 60 °C, 5 min at 95 °C, 175 min at 60 °C, and subsequently held to 20 °C in a PCR machine. The bisulfite-treated DNA solution was applied to EpiTect spin column according to manufacture’s protocol. Bisulfite-treated DNA was eluted in 20 μL of elution buffer.

### Bisulfite sequencing analysis

PCR was performed with HotStarTaq DNA Polymerase (Qiagen) for 35 cycles with primers listed in Supplementary Data Table S1. The PCR products were cloned using the PCR Cloning kit (Qiagen) according to the manufacturer’s instructions. Individual clones were isolated using the Exprep Plasmid SV mini kit (GeneAll) and sequenced using the T7 promoter primer. The sequence data were analyzed using QUMA (quantification tool for methylation analysis; http://quma.cdb.riken.jp).

### Karyotype analysis

Karyotype analysis was performed according to standard method at the Korea Research of Animal Chromosomes, Korea.

### Short tandem repeat (STR) analysis

Genomic DNA was used for PCR with PowerPlex STR kit (Promega). PCR was used to simultaneously amplify 16 STR loci across in each sample: DS81179, D21S11, D7S820, CSF1PO, D3S1358, TH01, D13S317, D16S539, D2S1338, D19S433, vWA, TPOX, D18S51, AMEL, D5S818, and FGA. Data analysis was performed with GeneMapper ID software version 3.2 (Applied Biosystems). STR analysis was performed according to the standard method at the genome analysis center, National Instrumentation Center for Environmental Management at Seoul National University, Korea.

### Single nucleotide polymorphism analysis of genomic homozygosity

Cell lines were genotyped with the Affymetrix Genome-Wide Human SNP Array 6.0 platform according to the manufacturer’s instructions. The SNP Array contains >1.8 million markers of genetic variation, >906,600 SNPs, and >946,000 probes for the detection of copy number variation; 1 μg of genomic DNA is needed for SNP genotyping.

## Supplementary information

Supplementary information.
