# Supplementary material for: Generation of human androgenetic induced pluripotent stem cells
Source: Sci Rep. 2020 Feb 27;10:3614. doi: 10.1038/s41598-020-60363-1 (PMC7046633; doi:10.1038/s41598-020-60363-1)
Supplement: Supplementary file 1 — Supplementary information. [file 41598_2020_60363_MOESM1_ESM.pdf]

Supplementary information

Generation of human androgenetic induced pluripotent stem cells

Na Young Choi[1,2], Jin Seok Bang[1,2], Yo Seph Park[3], Minseong Lee[1,2], Han Sung Hwang[4], Kisung Ko[5], Soon Chul Myung[6], Natalia Tapia[7], Hans R. Schöler[8,9], Gwang Jun Kim[10], Kinarm Ko[1,2,11,*]

[1]Department of Stem Cell Biology, School of Medicine, Konkuk University, Seoul 05029, Republic of Korea; [2]Center for Stem Cell Research, Institute of Advanced Biomedical Science, Konkuk University, Seoul 05029, Republic of Korea; [3]Department of Stem Cell Research, TJC Life Research and Development Center, TJC Life, Seoul 06698, Republic of Korea; [4]Department of Obstetrics and Gynecology, Research Institute of Medical Science, Konkuk University School of Medicine, Seoul 05030, Republic of Korea; [5]Department of Medicine, College of Medicine, Chung-Ang University, Seoul 06974, Republic of Korea; [6]Department of Urology, Chung-Ang University College of Medicine, Seoul 06974, Republic of Korea; [7]Institute of Biomedicine of Valencia, Spanish National Research Council, Jaime Roig 11, 46010 Valencia, Spain; [8]Department of Cell and Developmental Biology, Max Planck Institute for Molecular Biomedicine, 48149 Münster, Germany; [9]Medical Faculty, University of Münster, 48149 Münster, Germany; [10]Department of Obstetrics and Gynecology, Chung-Ang University Hospital, Chung-Ang University College of Medicine, Seoul 06973, Republic of Korea; [11]Research Institute of Medical Science, Konkuk University, Seoul 05029, Republic of Korea

[*]Correspondence: knko@kku.ac.kr

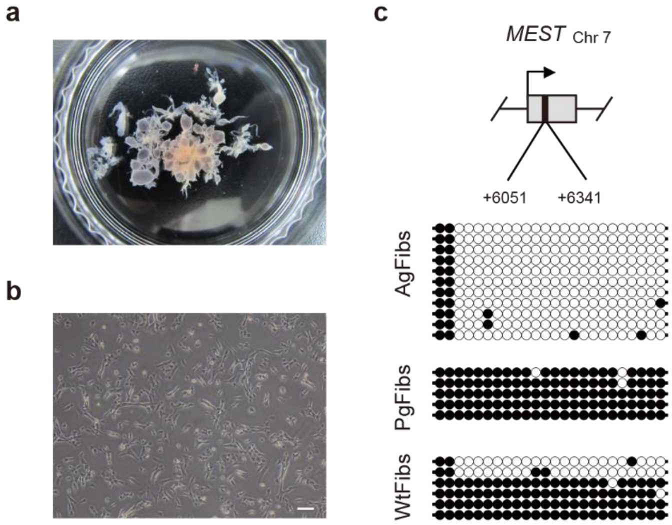

Supplementary Figure S1. DNA methylation analysis of the *MEST* gene. (**a**) Morphologically verified complete hydatidiform mole tissue. (**b**) Morphology of AgFibs. (**c**) DNA methylation status of *MEST* in AgFibs, PgFibs, and biparental fibroblasts (WtFibs) was analyzed by bisulfite sequencing. Each line represents a separate clone. Black and white circles represent hypermethylated and hypomethylated CpGs, respectively.

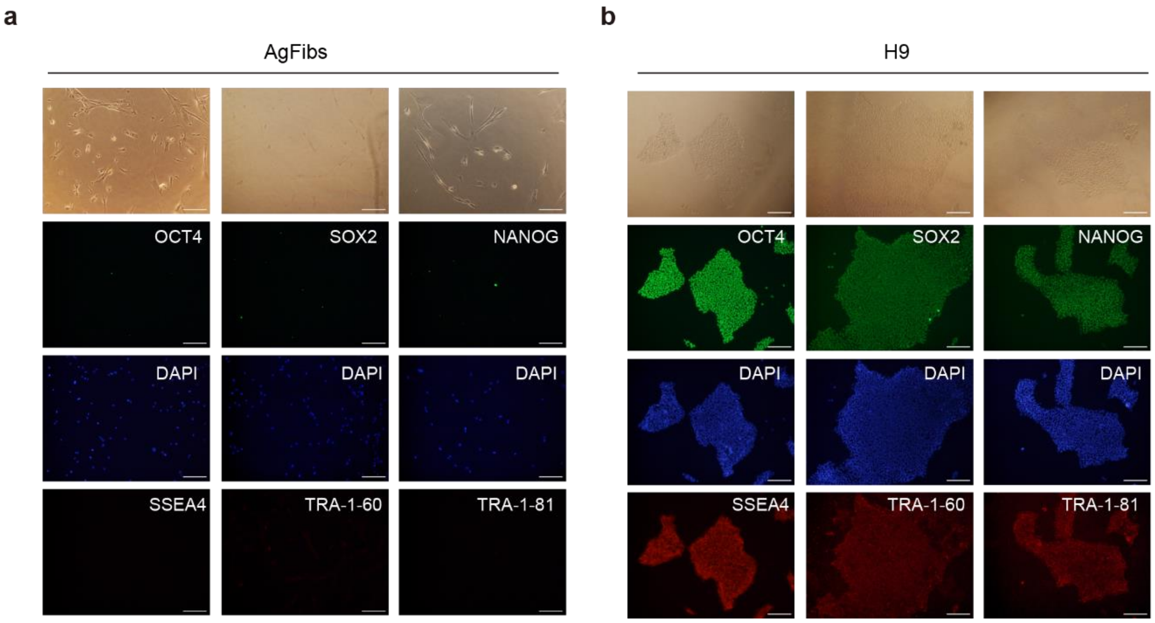

Supplementary Figure S2. Immunocytochemical analysis of pluripotency marker genes. Immunocytochemistry for pluripotency markers (OCT4, SOX2, NANOG, SSEA4, TRA-1-60, and TRA-1-81) in (**a**) AgFibs (negative control) and (**b**) H9 (positive control). Scale bars = 100 μm.

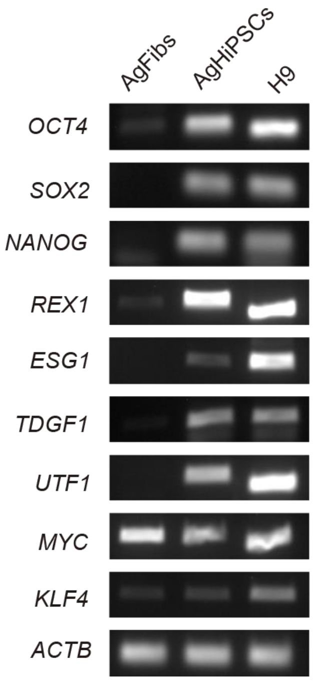

Supplementary Figure S3. Expression of pluripotency-specific maker genes. RT-PCR analysis of pluripotency-specific gene expression in AgFibs, AgHiPSCs, and H9. *ACTB* was used as a reference gene. The full-length gel image is presented in Supplementary Figure. S4

The full-length gel data

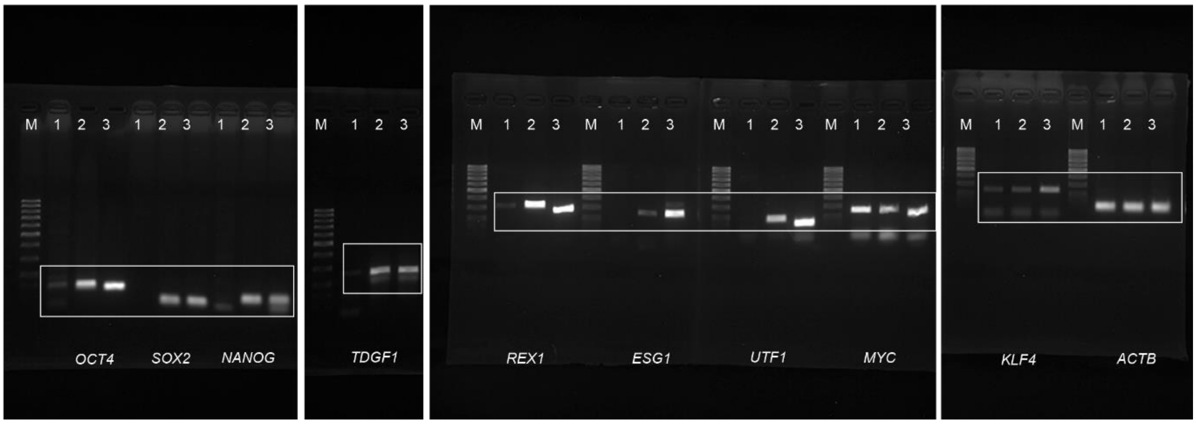

Supplementary Figure S4. Expression of pluripotency-specific maker genes. M, marker; 1, AgFibs; 2, AgHiPSCs; 3, H9.

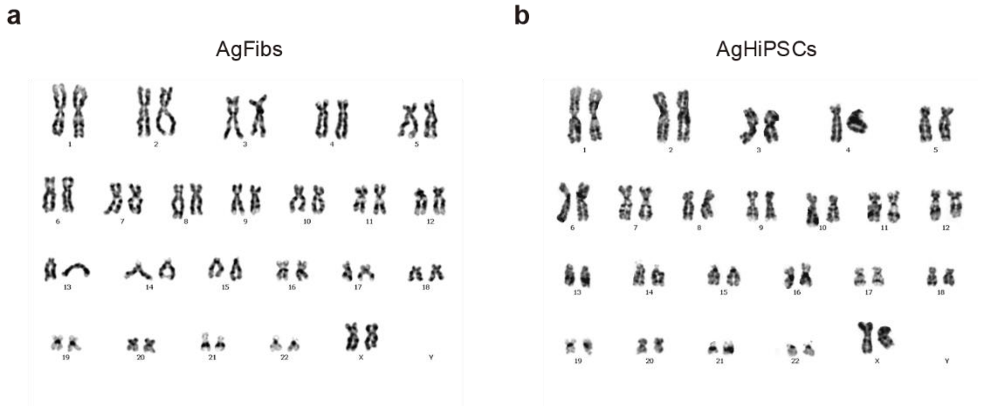

Supplementary Figure S5. Karyotyping of androgenetic cell lines. Confirmation of a normal 46, XX karyotype of (**a**) AgFibs and (**b**) AgHiPSCs.

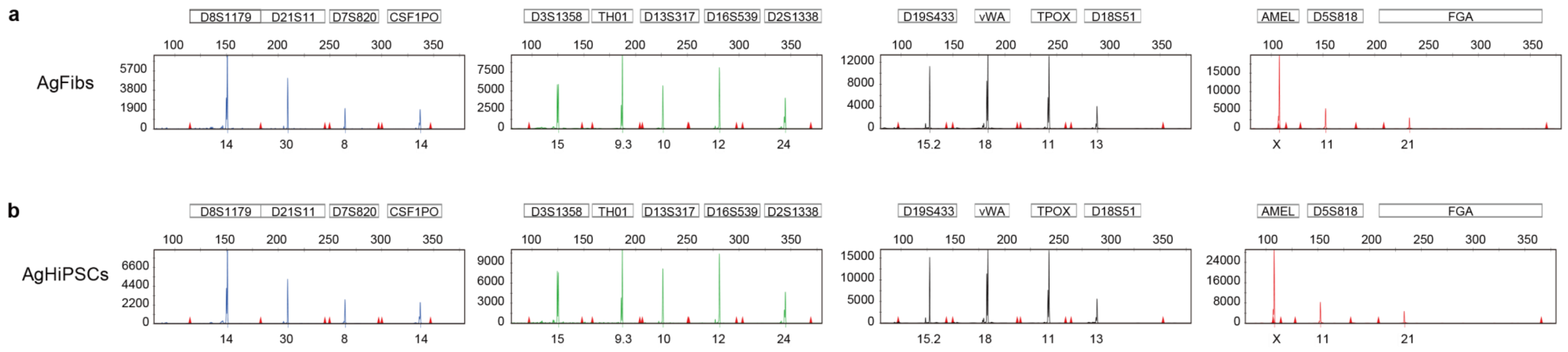

Supplementary Figure S6. Short Tandem Repeat (STR) profiling of (**a**) AgFibs and (**b**) AgHiPSCs. We confirmed the STR analysis that the AgFibs and AgHiPSCs are of the same origin. The peaks at each locus were converted to numerical values based on allelic ladders.

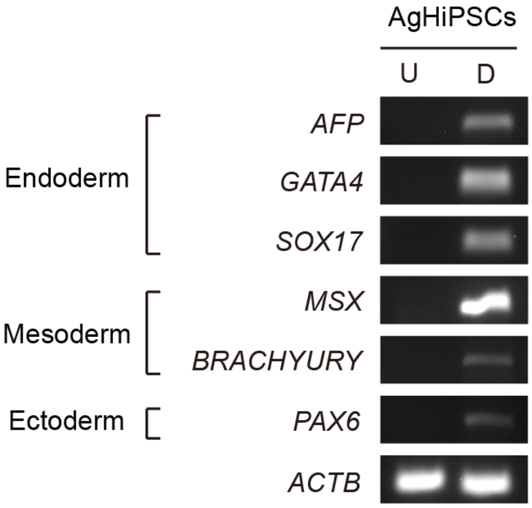

Supplementary Figure S7. *In vitro* differentiation of AgHiPSCs. RT-PCR analysis of the markers of three germ layers in embryoid bodies derived from AgHiPSCs. U, undifferentiated cells; D, differentiated cells. The full-length gel image is presented in Supplementary Figure. S8.

The full-length gel data

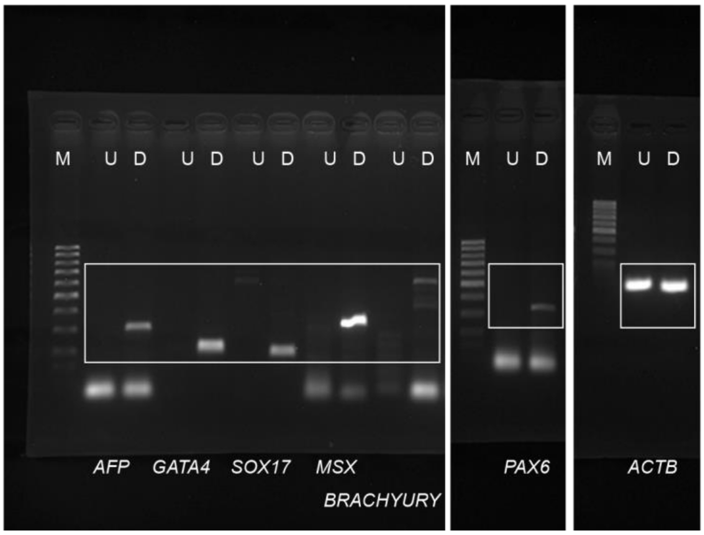

Supplementary Figure S8. *In vitro* differentiation of AgHiPSCs. M, marker; U, undifferentiated cells; D, differentiated cells.

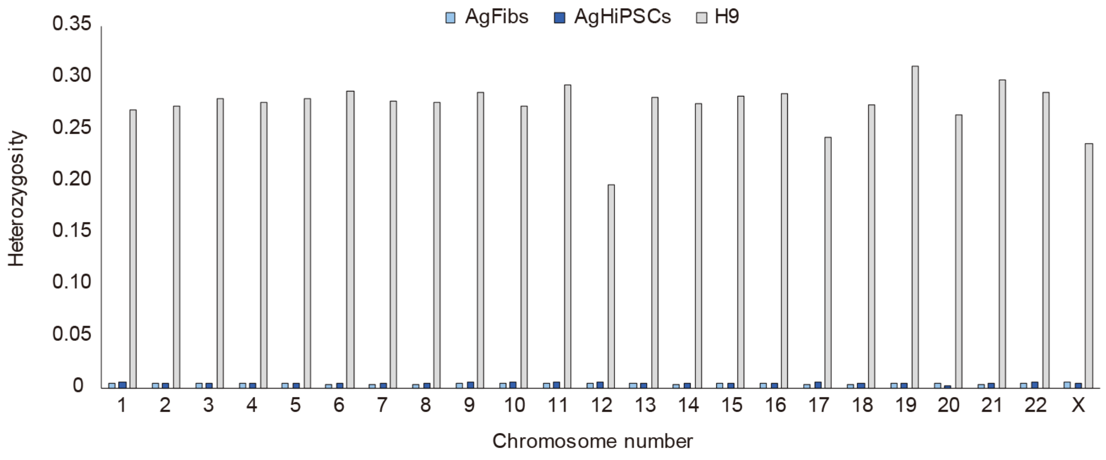

Supplementary Figure S9. Percentage of SNP heterozygote calls. Heterozygosity of AgFibs (0.46%) and AgHiPSCs (0.52%) was analyzed using a SNP array, and H9 cells (27.28%) served as a control.

Table S1. Sequence of primer sets used in bisulfite sequencing

Gene	Sequence
H19	F-AGG TGT TTT AGT TTT ATG GAT GAT GG
	R-TCC TAT AAA TAT CCT ATT CCC AAA TAA CC
MEST	F-TCG TTG TTG GTT AGT TTT GTA CGG TT
	R-AAA AAT AAC ACC CCC TCC TCA AAT
SNRPN	F-GAG GGA GGG AGT TGG GAT TTT TG
	R-ACC GCT CCC CAA ACT ATC TCT T
MAGEL2	F-AAG TTA ATT GGA GGT GGA TTT TAA G
	R-CCA ACT ATC AAC AAA AAA ACA TAT C
